# Do learning style preferences influence the cumulative gross point average and self directed learning hours in dental students: a preliminary study

**DOI:** 10.1186/s12909-022-03535-z

**Published:** 2022-06-24

**Authors:** Kiran Kumar Ganji, Mohammad Khursheed Alam, Ravi Kumar Gudipaneni, Hmoud Algarni, Manay Srinivas Munisekhar, May Osman Hamza, Mohammed Assayed Mousa, Mohammed Ghazi Sghaireen

**Affiliations:** 1 Department of Preventive Dentistry, College of Dentistry, Jouf University, Aljouf Province Sakaka, Saudi Arabia; 2 Center for Transdisciplinary Research (CFTR), Saveetha Dental College, Saveetha Institute of Medical and Technical Sciences, Saveetha University, Chennai, India; 3 Department of Operative Dentistry & Endodontics, College of Dentistry, Jouf University, Sakaka, KSA Saudi Arabia; 4 Department of Prosthetic Dental Sciences, College of Dentistry, Jouf University, Sakaka, KSA Saudi Arabia

**Keywords:** Learning styles, cumulative gross point average score, Self-direct learning, Dental students

## Abstract

**Background:**

Learning styles influence the outcome of the student performances based on preliminary data available. To evaluate whether the learning styles discriminate the cumulative gross point average (CGPA) scores and self-directed learning hours (SDL) in an integrated curriculum of dental students.

**Methods:**

Participants in this blinded cross-sectional study were dental students enrolled in the Bachelor of Dental & Oral Surgery program at XXXX College of Dentistry. An online survey (Kolb Learning Style Inventory) was used to collect data. It has four sections: Concrete Experience (CE), Abstract Conceptualization (AC), Reflective Observation (RO), and Active Experimentation (AE). Questionnaire was distributed electronically to students of Academic level 1 to 5, selected by using non-probability quota sampling technique. In addition to learning style assessment the questionnaire also included measures to obtain data such as gender, academic level, CGPA score, and SDL hours of participants. The CGPA scores were categorized into average (3 to 3.6), good (3.7 to 4.2), excellent (4.3 to 4.7) and outstanding (> 4.7) as well as SDL in to three classes as, < 1 h, > 1 but < 3 h and > 3 h. Discriminant function test was computed to assess the effectiveness of discrimination by the learning styles in GPA and SDL.

**Results:**

The study's questionnaire was completed by 198 participants (43% females and 57% males). Learning styles were discriminated by excellent category of CGPA scores that presented 72.1% group membership whereas in case of outstanding category presented the least as 17% group membership. Learning styles were discriminated by > 2 but < 3 h category of SDL hours that presented 69.7% group membership.

**Conclusion:**

Learning styles can be used to discriminate the student academic performances and self-directed learning hours. Among the different category of CGPA participants with outstanding performance represent a good prediction for learning styles preferences. Participants with varying SDL hours also influenced the learning styles.

## Introduction

Learners' learning styles have been defined as a multi-step process that requires them to take in information, process it, preserve it, and then remember it [1]. Recognizing each student's unique learning style may be quite beneficial in the educational process as a whole. The current trend in education is away from pedagogy and toward andragogy, or learning based on students centred rather than teachers centered [2, 3]. It can be difficult for teachers to efficiently convey material to students if their teaching strategies do not fit the students' learning styles [4]. There are several ideas on how people learn, but one of the most popular is called experiential learning theory (ELT) [5]. As per ELT proposed by David Kolb, two dialectically grasping modes were Concrete Experience (CE) and Abstract Conceptualization (AC) as well as Reflective Observation (RO) and Active Experimentation (AE) were two dialectically transforming experience that focus on experience as the important factor to the learning process [6]. In contrast to other learning theories, ELT emphasizes the important significance of experience [7, 8]. Hosseini et al. argued that while a dominant learning style is important in predicting educational performance, teachers should also consider students' learning preferences when developing learning opportunities [9]. Students and teachers who are aware of their learning styles are more likely to succeed in school [10].

The Cumulative Grade Point Average (CGPA) is used to assess students' academic achievement. Many factors, such as gender, previous academic performance, living place and income level of family, social environment, time spent studying, learning ability, and living place during university life, can act as both a barrier and a catalyst to students achieving a high CGPA that reflects their overall academic performance.

SDL's philosophy, which encompasses theories of adult education, humanism, constructivism, and empowerment, resonates well with the Schön and Kolb learning models [12, 13]. According to Knowles [11] self-directed learning (SDL) is an entity in which individuals take the initiative in assessing their own learning requirements, establish learning objectives, and discover human and material resources. They then choose and apply suitable learning techniques and evaluate the results. Preclinical students may benefit from self-directed learning in an integrated curriculum, according to Kidane et al., who cited PBL as a component of the hybrid curriculum [14]. With a strong positive link between self-directed learning readiness and learning presence, Hwang et al. found that 71% of what students learned was transferred to the real world [15].

Despite the absence of proof that learning styles are really beneficial in assessing students' academic success, a recent analysis confirms that current research articles 'about' Learning Styles in higher education that strongly support their usage [16]. As a result, the majority of data was based on assumptions rather than testing learning styles directly. For example, researchers got learning styles questionnaire completed by the group of students and then make recommendations that are implemented based on the results to reform the curriculum. Then later on whether the reformed curriculum based on learning styles preferences would actually promote the academic performance of the students and determination of which learning style based instruction is so often anticipated to have merit was not tested.

Sufficient evidence exists in providing the relationship and importance of SDL and preferred learning styles of various fields but current literature doesn’t show any studies that investigated the effectiveness of corresponding level of learning styles as per KOLB. The researchers are interested to know whether KOLB model of learning style preferences discriminate the CGPA and self-directed learning hours of the dental students.

## Materials and methods

In the present cross-sectional study the participants were the dental students cohort involved in Bachelor of Dental & Oral Surgery program for the academic year 2020–21 at College of Dentistry, Jouf University, KSA. All methods were performed according to relevant guidelines. Participants remained non-anonymous with voluntary participation in the study. An email invitation was sent to all eligible participants, asking them to complete the survey. This entailed sending an invitation to participate and then only sending the questionnaire to those who responded positively to the invitation and provided consent. This was an extra step in survey recruitment that required potential participants to give consent to be invited to participate in a survey, and then to give passive consent by returning the completed copy upon receipt of the questionnaire. Questionnaire was distributed electronically to students using non-probability quota sampling technique. Students from the academic level 1 to 5 enrolled for the program were included in this study. The students enrolled in specific courses of the said program wherein SDL is not a part of the course learning activities were excluded from the study. Data collection was done using a questionnaire (Kolb Learning Style Inventory version 3.1) [17] consisting of 4 separate sections. Each section represented a particular learning style of KOLB model such as concrete experience, reflective observation, abstract conceptualization and active experimentation. In total the questionnaire contained 80 questions which were equally distributed to represent all the four learning styles of KOLB model. The questionnaire included instructions and a guide to help the participants to get notified of results and also calculation of their learning style based on the results of the responses. Provision was made to generate the strength of learning style which was assessed on scale ranging from 1 to 5, graded as 1- very low preference, 2- low preference, 3-Moderate preference, 4- strong preference and 5- very strong preference.In addition to learning style assessment the questionnaire also included measures to obtain data such as gender, academic level, CGPA score, and SDL hours of participants. The participants were advised to input the CGPA score from their individual portal account of academic performance provided by the university and regarding the number of SDL hours employed by the students during the current course. This study was conducted according to the Local committee of Bioethics guidelines of the Jouf University, KSA. The Local committee of Bioethics granted ethical clearance for this project (14–15-9/40). The content validity of the questionnaire items assessing learning styles were checked by two senior teaching staff members not involved in the study and were found to have very good internal consistency (Cronbach’s alpha score 0.89). The realibility index for the four learning modes of KOLB model were: concrete experience α = 0.89, reflective observation α = 0.82, abstract conceptualization α = 0.86 and active experimentation α = 0.81.

### Statistical analysis

IBM SPSS Statistics for Windows® (version 23.0, IBM Corp, Armonk, NY) was used for data entry and statistical analyses. For analytic reasons, the CGPA score of each student released by university was standardized using visual binning function in SPSS and into four academic performance categories by values (with three cut-off points). The four academic performance categories for CGPA scores were average (3 to 3.6), good (3.7 to 4.2), excellent (4.3 to 4.7) and outstanding (> 4.7). Similarly the SDL hours were also categorized in to three classes as SDL hours < 1 h, > 1 but < 3 h and > 3 h. Discriminant function test was computed to assess the effectiveness of discrimination by the learning styles in GPA and SDL.

## Results

A total of 198 participants responded to the questionnaire of the study. Out of 198 participants 43% were female and 57% were males. The mean preferences scores of learning styles in relation to different groups of CGPA scores are presented in Table 1. AE was preferred learning style among all students with different CGPA scores. The mean preferences scores of learning styles in relation to different groups of SDL hours are presented in Table 2. RO was the least preferred learning style among all the student with different level of SDL hours. RO & CE were the only 2 learning styles that demonstrated significant difference (*p* < 0.05) with respect to CGPA scores suggesting that RO & CE differ with varying CGPA scores of the participants (Table 3 & 4). Table 5 represent preferences of learning styles of all participants over different CGPA scores and SDL hours in order of ranking. AE was the most preferred learning style among all levels of CGPA scores and SDL, whereas RO, AC & CE learning styles varied for different ranking with different level of CGPA and SDL hours. Learning styles were discriminated by excellent category of CGPA scores that presented 72.1% group membership whereas in case of outstanding category presented the least as 17% group membership (Table 6). Learning styles were discriminated by > 2 but < 3 h category of SDL hours that presented 69.7% group membership (Table 7).

**Table 1:** Mean preferences scores of learning styles with different group of CGPA scores (*n* = 198)

CGPA score	Learning style preferences	Mean	Std. Deviation
Average (*n* = 50)	RO	2.96	.98
	AC	3.01	.98
	CE	3.40	1.27
	AE	3.84	.82
Good (*n* = 46)	RO	2.95	.93
	AC	2.83	1.20
	CE	2.61	1.24
	AE	4.00	.91
Excellent (*n* = 49)	RO	3.33	1.34
	AC	3.27	1.25
	CE	3.86	1.28
	AE	4.25	.79
Outstanding (*n* = 53)	RO	3.59	1.03
	AC	2.85	1.25
	CE	3.10	1.22
	AE	4.23	.66

**Table 2:** Mean preferences scores of learning styles with different group of SDL hours

SDL	Learning style preferences	Mean	Std. Deviation
< 1 h	RO	3.08	1.17
	AC	3.14	1.22
	CE	3.50	1.31
	AE	4.26	.64
> 2 but < 3 h	RO	3.16	1.12
	AC	2.88	1.20
	CE	3.19	1.37
	AE	4.08	.90
> 3 h	RO	3.49	1.10
	AC	3.11	1.12
	CE	3.33	1.27
	AE	3.88	.82
	AC	3.02	1.18
	CE	3.33	1.33
	AE	4.09	.81

**Table 3:** Tests of Equality representing Wilks’ Lamba for individual means of learning styles in CGPA and SDL

Learning styles	Wilks' Lambda		F		df1		df2		Sig	
	CGPA	SDL	CGPA	SDL	CGPA	SDL	CGPA	SDL	CGPA	SDL
RO	.948	.981	3.748	2.039	3	2	205	206	.012*	.133
AC	.975	.989	1.757	1.107	3	2	205	206	.157	.333
CE	.882	.990	9.159	1.084	3	2	205	206	.000*	.340
AE	.956	.969	3.181	3.327	3	2	205	206	.025	.038

* statistically significant *p*<0.05

**Table 4:** Box’s statistics of learning styles in relation to CGPA and SDL

Box’s Test of equality		Learning styles Vs CGPA	Learning styles Vs SDL
Box's M		37.572	20.541
F	Approx	1.207	.997
	df1	30	20
	df2	94,150.563	110,765.142
	Sig	.001	.000

**Table 5:** Fisher's linear discriminant functions for CGPA and SDL based on learning styles

Learning styles	CGPA				SDL		
	Average	Good	Excellent	Outstanding	< 1 h	> 2 but < 3 h	> 3 h
RO	2.249^2^	2.217^2^	2.554^2^	2.777^2^	1.926^2^	2.021^2^	2.272^2^
AC	1.384^4^	1.388^3^	1.456^4^	1.225^4^	1.496^3^	1.337^3^	1.438^3^
CE	1.479^3^	.955^4^	1.722^3^	1.298^3^	1.155^4^	1.036^4^	1.128^5^
AE	5.688^1^	6.027^1^	6.270^1^	6.335^1^	6.107^1^	5.856^1^	5.499^1^
(Constant)	-20.26	-20.14	-24.42	-23.65	-21.52	-19.61	-20.14

^1,2,3,4 and 5^ represents the ranking order of the values to be used in prediction of learning styles for each variable of CGPA and SDL

**Table 6:** Classification results of CGPA for predicted group membership with learning styles as constraints

CGPA scores	Predicted group membership (%)				Total (%)
	Average	Good	Excellent	Outstanding	
Average	23.1*	21.2	48.1	7.7	100
Good	28.6	33.3*	21.4	16.7	100
Excellent	7.4	11.8	72.1*	8.8	100
Outstanding	12.8	17.0	53.2	17.0*	100

* statistically significant *p*<0.05

**Table 7:** Classification results of SDL for predicted group membership with learning styles as constraints

SDL	Predicted Group Membership (%)			Total (%)
	< 1 h	> 2 but < 3 h	> 3 h	
< 1 h	22.4*	70.1	7.5	100
> 2 but < 3 h	21.3	69.7*	9.0	100
> 3 h	13.2	73.6	13.2*	100

* statistically significant *p*<0.05

## Discussion

Learning styles are thought to have a minor impact on academic performance. Regardless of how minor the effect on learning outcomes, it is widely acknowledged that learning styles can help students improve their own learning and thus encourage self-directed learning. The results of present study showed that learning styles did influence and discriminated CGPA scores of participants with very much influence by RO and AE learning styles. The model fit that learning style discriminating excellent category of CGPA participants was more predictable (Predicted group membership 72%) in comparison with other category of CGPA participants. Other category of CGPA such as Good, Average and Outstanding were discriminated by learning style preferences but the prediction percentage was very low. Kies argues that a student's learning style contributes to their disparities in performance [18]. Ghaffari et al. found no correlation between students' learning styles and academic success in medical students, with accommodator and converger learning styles being the most favoured [19]. According to Torres Martinez et al., active and reflective learning styles help students develop the dental skills needed to face clinical scenarios, and students adapt to learning through various strategies and have the capacity to adapt to various situations, which facilitate learning in dentistry [20]. The reason for such variations in preferences of learning can be attributed to traditional and integrated curriculum within the dental colleges of Kingdom of Saudi Arabia [4, 21]. Students and interns in Saudi Arabia used the Kolb's concept of diverging learning styles, with ALQahtani and AlGahtani finding this to be the most prevalent. They also found no link between learning style preferences and the cumulative gross point scale (CGPA) [21]. Integrated curriculum introduced in medicine field is well supported and accredited by international organizations [22, 23]. The integrated dentistry curriculum offers the opportunity to organize information from many disciplines around a common subject, as well as to accommodate students of various learning styles. Dentistry program at College of Dentistry, Jouf University (KSA) is a five-year integrated dental program, in which the foundational knowledge is dealt largely in first two preclinical years whereas focusing on clinical skills and practice in the third, fourth & fifth years. Students are exposed to clinical experience at various points throughout their education. Lectures, problem-based learning, case-based learning, small-group discussions, laboratory work, and clinical practice under supervision are various methods of delivering the curriculum. As a rule, assessments are done in accordance with the instructional strategies, such as by using a written format and/or an objective structured practical/clinical examination/assignment combination. Active, self-directed, and experiential learning are all part of the curriculum's goals.

Students have found that active learning helps them retain information, improves their interpersonal skills, develops their critical thinking, and keeps them motivated to continue their education [24, 25]. Our findings showed that in the CGPA group most of the participants preferred AE and CE learning styles who chosen to learn by forming intellections, which are in consistent with the results of some studies conducted among dental, medical and nursing students which were predictable in biological sciences [26–28].

In the current study participants with SDL hours (> 2 but < 3 h) preferred AE method of learning style as wells as these (> 2 but < 3 h) discriminated the learning styles. These findings were in agreement with the study done on pediatric residents of medical institute who reported that the more advanced the residents were in their training, the more they used SDL resources [29]. According to Peng, a self-directed group (less than 30% didactics, open library access, self-study and group discussions) and a control group were randomly assigned to medical schools in China (didactics, limited library access). When it came to fundamental knowledge exams in Inorganic Chemistry, Biochemistry, and Microbiology, as well as applied knowledge in Human Anatomy and overall biochemical knowledge, students in the SDL group performed considerably better than their peers [30, 31]. As documented by Abraham et al., students in a self-directed course in physiology, which included presentations and group discussions led by medical students, scored considerably higher on exams during SDL sessions than they did during lecture sessions [32]. One of the factor that supports our findings in the current study is related to key principles of SDL where in educator as a facilitator which is followed in curriculum of our dental school. The facilitator identifies the learning needs and develops the objectives which is an integral component of SDL. According to Knowles, successful learning is more likely when learners clearly define their learning requirements and their needs are in harmony with social, organizational, or academic objectives [11]. There is really no high-quality data to establish the qualities of learners who are most suited for SDL. If you are an adult learner with a reservoir of information and can apply what you've learned right away to your practice, this method may be more suited for you, according to Knowles and others [33].

Recently, many dental schools in Kingdom of Saudi Arabia have started integrated core syllabus in their dental programs. For educators, not only using an operational, competent and suitable learning style for their country, but also knowing usefulness of learning style predictions in academic achievement and identifying the duration of self-directed learning amongst students are crucial features of a top-notch dentistry schooling system. Current study is one of the first study that evaluates discrimination of CGPA and self-directed learning hours of the dental students in integrated dental curriculum by learning style preferences. Furthermore, one of the strengths of the study is that it explains the influence of various learning styles in CGPA and SDL hours of dental students, as major key points in designing the program outcome learning outcomes in an competency based dental curriculum.

Drawback of the study include that, due to the nature of convenience sampling the participants who participated in the study belong to a structured curriculum pattern with vertical integration which may not be representative of other traditional curriculum pattern; hence, the results cannot be generalized. Meanwhile, the KOLB inventory learning styles were self-reported, which may have resulted in bias. Our study, like all others, has sparked new ideas for further investigation. These might include the following: Does learning styles should be taken in consideration in designing the teaching methodology of the course to be in concurrent with teaching and learning process. What is the trajectory of SDL in assessing the learning styles of dental students. And most importantly, how can educators promote importance of learning styles in designing an dental curriculum for future health professionals?

## Conclusion

Learning styles can be used to discriminate the student academic performances with respect to CGPA scores and self-directed learning hours. Among the different category of CGPA, participants with outstanding performance represent a good prediction for learning styles preferences. Participants with varying SDL hours also influenced the learning styles.

## Data Availability

The data will be available upon request from the corresponding author.

## References

[1] Jiraporncharoen W, Angkurawaranon C, Chockjamsai M, Deesomchok A, Euathrongchit J (2015). Learning styles and academic achievement among undergraduate medical students in Thailand. J Educ Eval Health Prof.

[2] Bennadi D, Kashinath K, Bharateesh J, Kshetrimayum N (2015). Assessing learning preferences of dental students using visual, auditory, reading-writing, and kinesthetic questionnaire. J Indian Assoc Public Health Dentistry.

[3] Ganji KK (2017). Evaluation of reliability in structured viva voce as a formative assessment of dental students. J Dent Educ.

[4] Asiry MA (2016). Learning styles of dental students. The Saudi J Dental Res.

[5] Kolb DA (1984). Experience as the source of learning and development.

[6] Kolb DA (2007). The Kolb learning style inventory.

[7] Armstrong E, Parsa-Parsi R (2005). How can physicians’ learning styles drive educational planning?. Acad Med.

[8] Bransford J, Brown A, Cocking R, Donovan S. How People Learn Brain, Mind, Experience, and School (Expanded Edition). In: Washington. DC: National Academy Press; 2000.

[9] Hosseini SM, Amery H, Emadzadeh A, Babazadeh S. Dental Students’ Educational Achievement in Relation to Their Learning Styles: A Cross-Sectional Study in Iran. Glob J Health Sci. 2015;7(5):152–8. 10.5539/gjhs.v7n5p152 PMC4803897 26156915

[10] Lubawy WC (2003). Evaluating teaching using the best practices model. Am J Pharm Educ.

[11] Knowles MS (1975). Self-directed learning: A guide for learners and teachers.

[12] Borduas F, Gagnon R, Lacoursière Y, Laprise R. The longitudinal case study: From Schön’s model to self-directed learning. J Contin Educ Health Prof. 2001;21(2):103–9. 10.1002/chp.1340210207 11420864

[13] Slotnick H (1999). How doctors learn: physicians’ self-directed learning episodes. Acad Med.

[14] Kidane HH, Roebertsen H, van der Vleuten CP (2020). Students’ perceptions towards self-directed learning in Ethiopian medical schools with new innovative curriculum: a mixed-method study. BMC Med Educ.

[15] Hwang S (2020). Differences in Self-Directed Learning Readiness, Learning Presence and Learning Transfer between Low-Achievers Participating in Peer Tutoring. The Journal of the Korea Contents Association.

[16] Newton PM (2015). The learning styles myth is thriving in higher education. Front Psychol.

[17] Kolb A. The Kolb learning style inventory versions 3.1 & 3.2 2013 technical specifications. In: Case Western Reserve University ; 2013. https://learningfromexperience.com.

[18] Kaplan EJ, Kies DA. Teaching styles and learning styles: Which came first? J Instructional Psychol. 1995;22:29–33.

[19] Ghaffari R, Ranjbarzadeh FS, Azar EF (2013). The analysis of learning styles and their relationship to academic achievement in medical students of basic sciences program. Res Develop in Med Educ.

[20] Ikeshoji EAB, de Lima Terçariol AA. Estilos de Aprendizagem: evidências a partir de uma revisão sistemática da literatura. Revista Diálogo Educacional. 2020;20(64):23–49.

[21] ALQahtani DA, Al-Gahtani SM (2014). Assessing learning styles of Saudi dental students using Kolb’s Learning Style Inventory. J Dent Educ.

[22] Chan W, Ng C, Yiu B (2006). A survey on the preference for continuing professional dental education amongst general dental practitioners who attended the 26th Asia Pacific Dental Congress. Eur J Dent Educ.

[23] Brauer DG, Ferguson KJ (2015). The integrated curriculum in medical education: AMEE Guide No. 96. Med Teach.

[24] Prince M (2004). Does active learning work? A review of the research. J Eng Educ.

[25] Gross CD (2019). Annotated bibliography: active learning, student resilience.

[26] Engleberg NC, Schwenk T, Gruppen LD (2001). Learning styles and perceptions of the value of various learning modalities before and after a 2nd-year course in microbiology and infectious diseases. Teach Learn Med.

[27] Schroder H, Henke A, Stieger L (2017). Influence of learning styles on the practical performance after the four-step basic life support training approach - An observational cohort study. PLoS ONE.

[28] El-Gilany A-H, Abusaad FES (2013). Self-directed learning readiness and learning styles among Saudi undergraduate nursing students. Nurse Educ Today.

[29] Dinkevich E, Ozuah PO (2003). Self-directed learning activities of paediatric residents. Med Educ.

[30] Wen-wei P (1989). Self-directed learning: a matched control trial. Teach Learning Med: Int J.

[31] Atta IS, Alghamdi AH (2018). The efficacy of self-directed learning versus problem-based learning for teaching and learning ophthalmology: A comparative study. Adv Med Educ Pract.

[32] Abraham RR, Upadhya S, Ramnarayan K (2005). Self-directed learning. Adv Physiol Educ.

[33] Malau-Aduli B, Smith A, Heggarty P, Teague P-A. A case for promoting self-directed learning among GP registrars. Townsville: Jamescook University; 2019.

